# Pien Tze Huang Inhibits Hypoxia-Induced Angiogenesis via HIF-1***α***/VEGF-A Pathway in Colorectal Cancer

**DOI:** 10.1155/2015/454279

**Published:** 2015-01-14

**Authors:** Hongwei Chen, Jianyu Feng, Yuchen Zhang, Aling Shen, Youqin Chen, Jiumao Lin, Wei Lin, Thomas J. Sferra, Jun Peng

**Affiliations:** ^1^Academy of Integrative Medicine, Fujian University of Traditional Chinese Medicine, 1 Huatuo Road, Minhou Shangjie, Fuzhou, Fujian 350122, China; ^2^Fujian Key Laboratory of Integrative Medicine on Geriatric, Fujian University of Traditional Chinese Medicine, 1 Huatuo Road, Minhou Shangjie, Fuzhou, Fujian 350122, China; ^3^Rainbow Babies & Children's Hospital, Case Western Reserve University School of Medicine, 11100 Euclid Avenue, Cleveland, OH 44106, USA; ^4^Postdoctoral Workstation, Zhangzhou Pien Tze Huang Pharmaceutical Co., Ltd., Shangjie, Zhangzhou, Fujian 363000, China

## Abstract

Hypoxia-induced angiogenesis plays an important role in the development and metastasis of solid tumors and is highly regulated by HIF-1*α*/VEGF-A pathway. Therefore, inhibiting tumor angiogenesis via suppression of HIF-1*α*/VEGF-A signaling represents a promising strategy for anticancer treatment. As a traditional Chinese medicine formula, Pien Tze Huang (PZH) has long been used as a folk remedy for cancer in China and Southeast Asia. Previously, we reported that PZH inhibits colorectal cancer (CRC) growth both in vivo and in vitro. To elucidate the antitumor mechanisms of PZH, in the present study we used human umbilical vein endothelial cells (HUVEC) and colorectal carcinoma HCT-8 cells to evaluate the effects of PZH on hypoxia-induced angiogenesis and investigated the underlying molecular mechanisms. We found that PZH could inhibit hypoxia-induced migration and tube formation of HUVEC cells in a dose-dependent manner, although the low concentrations of PZH had no effect on HUVEC viability. Moreover, PZH inhibited hypoxia-induced activation of HIF-1*α* signaling and the expression of VEGF-A and/or VEGFR2 in both HCT-8 and HUVEC cells. Collectively, our findings suggest that PZH can inhibit hypoxia-induced tumor angiogenesis via suppression of HIF-1*α*/VEGF-A pathway.

## 1. Introduction

Colorectal cancer (CRC) is the third most commonly diagnosed cancer worldwide and the second leading cause of cancer mortality in the developed world [1]. Approximately one million new cases are diagnosed every year and 500,000 deaths are reported annually [2]. CRC is a disease characterized by activation of multiple signaling pathways which stimulate proliferation and angiogenesis as well as invasion and metastasis [3–5]. During the last decade, the survival rate of CRC has increased due to early diagnosis, staging, and improvements in surgery and therapy [6, 7]. However, tumor progression to a highly metastatic stage (mCRC) decreases the overall 5-year survival rate to below 10%, in part due to tumor angiogenesis, which is required for tumor growth and metastasis [8–11].

Angiogenesis is a central mechanism in human colorectal cancer development and growth [12, 13]. In particular, vascular endothelial growth factor (VEGF) is closely associated with the induction of neovascularization in human colon cancer [14]. VEGF is one of the most important endogenous ligands of receptors present on the endothelial cell plasma membrane and its binding leads to intracellular signaling and, ultimately, gene transcription that promotes endothelial proliferation, migration, and tube formation of endothelial cells resulting in tumor growth and metastasis [15, 16]. In addition, VEGF is mediated by multiple interacting genetic and environmental signals [17]. A hypoxic microenvironment that is critical for the tumorigenic process in general can stimulate VEGF via hypoxia inducible factor-1 (HIF-1), its primary regulator during hypoxic conditions.

HIF-1 is a dimeric transcription factor composed of the HIF-1*α* and HIF-1*β* subunits. HIF-1*α* is a key player in the maintenance of cellular homeostasis under hypoxic conditions through its regulation of the expression of many genes involved in a crucial aspect of cancer biology [18–20]. Under normoxic conditions, prolyl hydroxylase mediates the hydroxylation of the proline residues of HIF-1*α* at two positions that allow HIF-1*α* to interact with the von Hippel-Lindau E3 ligase complex which allows for degradation by the ubiquitin-proteasome system [21]. Under hypoxic conditions, HIF-1*α* is not degraded and is able to bind with the ubiquitously expressed HIF-1*β* subunit. The heterodimeric HIF-1 rapidly translocates to the nucleus, where it binds to the hypoxia-responsive element and regulates several genes responsible for cellular development under hypoxic conditions. One of the HIF-1-responsive genes is VEGF, which promotes angiogenesis in tumors [22].

Owing to the important role of angiogenesis in the progression and metastasis of solid tumors, inhibition of tumor angiogenesis has become a promising strategy for anticancer chemotherapy. Various kinds of antiangiogenic agents are currently being developed. However, redundancy and cross talk in these pathways form a complicated and robust network which is difficult to target. Currently, angiogenesis inhibitors that target only a single pathway may be insufficient and may contribute to drug resistance [23]. These problems highlight the need for the development of novel anticancer agents. Natural products, such as TCM, have been used as therapies for a very long time [24–26]. Pien Tze Huang (PZH) is a well-known TCM formula that was first prescribed 450 years ago in the Ming Dynasty. PZH has been used in China and Southeast Asia for centuries as a folk remedy for various types of cancer [27, 28]. We have previously reported that PZH can inhibit colon cancer cell growth in vitro via promotion of apoptosis [29]. In addition, using a CRC mouse xenograft model, we found that PZH can suppress tumor growth in vivo without apparent adverse effects and was shown to inhibit vascular endothelial cell proliferation in vitro and capillary tube formation in vivo [30, 31]. The effects of PZH on hypoxia-induced angiogenesis during hypoxic conditions have not been explored.

Here, we show that PZH strongly downregulates the expression of HIF-1*α* and inhibits angiogenesis in hypoxic conditions. PZH potently inhibited hypoxia-induced transcription and translation of VEGF-A. Moreover, we found that PZH ameliorates hypoxia-stimulated angiogenesis. These results demonstrate that PZH may inhibit hypoxia-induced angiogenesis by downregulating HIF-1*α*/VEGF-A activity.

## 2. Materials and Methods

### 2.1. Materials and Reagents

All chemicals, unless otherwise stated, were obtained from Sigma-Aldrich (St. Louis, MO, USA). Roswell Park Memorial Institute Medium 1640 (RPMI RPMI-1640), penicillin-streptomycin, fetal bovine serum (FBS), Trypsin-EDTA, and TRIzol reagent were purchased from Invitrogen (Carlsbad, CA, USA). SuperScript II reverse transcriptase was obtained from Promega (Madison, WI, USA). In vitro angiogenesis assay kit was purchased from Millipore (Billerica, MA, USA). Enzyme linked immune sorbent assay (ELISA) kits of human VEGF-A were obtained from Shanghai Xitang Biological Technology Ltd. (Shanghai, China). HIF-1*α*, *β*-actin antibodies, and horseradish peroxidase- (HRP-) conjugated secondary antibodies were purchased from Cell Signaling (Beverly, MA, USA).

### 2.2. Preparation of PZH

PZH was obtained from and authenticated by the sole manufacturer Zhangzhou Pien Tze Huang Pharmaceutical Co., Ltd., China (Chinese FDA Approval number Z35020242). Stock solution of PZH was prepared immediately prior to use by dissolving the PZH powder in phosphate buffered saline (PBS) to a concentration of 20 mg/mL.

### 2.3. Cell Culture

Human umbilical vein endothelial cells (HUVECs) and human colon carcinoma cells (HCT-8) were obtained from Nanjing KeyGen Biotech. Co. Ltd. (Nanjing, Jiangsu, China). HCT-8 and HUVECs cells were grown in RPMI-1640 containing 10% (v/v) FBS and 100 U/mL penicillin and 100 *μ*g/mL streptomycin. Cells were cultured at 37°C, in a 5% CO_2_ humidified environment (normoxic). To expose the cells to hypoxia, they were placed in a modular incubator chamber (Thermo Forma, Waltham, MA, USA), which was infused with a mixture of 1% O_2_, 5% CO_2_, and 94% N_2_.

### 2.4. Cell Viability Evaluation

Viability of HUVECs was carried out using a 3-(4,5-dimethylthiazol-2-yl)-2, 5-diphenyltetrazolium bromide (MTT) assay. Briefly, HUVECs were harvested from exponential phase cultures growing in RPMI-1640 supplemented with 10% FBS, counted, plated in 96-well flat-bottomed microtiter plates (100 *μ*L cell suspensions, 6 × 10^4^ cells/mL for HUVECs cells), and treated with medium containing various concentrations of PZH. After 24 hours, the media were removed and 100 *μ*L of MTT solution (0.5 mg/mL in PBS) was added, and the reaction mixture was incubated at 37°C in a 5% CO_2_ atmosphere for 4 hours. The MTT solution was removed and 100 *μ*L of DMSO added. Optical density was measured using a spectrophotometer (Bio-Tek Model ELX800, USA) at 570 nm.

### 2.5. Wound-Healing Cell Migration Assay

HUVECs were plated at 4 × 10^5^ cells/well on a 6-well plate in normal culture media and allowed to reach 80–90% confluency. An injury line with a width of 0.6~1.0 mm was made with a sterile pipette tip and cells were rinsed with PBS. The cells were incubated with serum-free medium supplemented with PZH (0.0625, 0.125, and 0.25 mg/mL) or medium alone as a control in hypoxia (1% O_2_) or normoxia (21% O_2_). Wound closure was evaluated 12 hours later with a Leica DMIL inverted microscope (Leica, German).

### 2.6. Migration Assay

Cell migration assays were performed with transwell cell culture chambers with 8 *μ*m pore filters (Corning, NY, USA). After treatment with various concentrations of PZH under normoxia for 24 hours, HUVECs cells were trypsinized and resuspended in serum-free RPMI 1640. HUVECs (5 × 10^4^ cells in 200 *μ*L of serum-free RPMI 1640) were added to the upper compartment in duplicate filters and RPMI-1640 media containing 10% (v/v) FBS were used as a chemoattractant in the lower chambers. Cells were allowed to migrate for 12 hours in normoxic/hypoxic conditions. The nonmigrating cells were removed from the upper surface of each transwell by cotton swab. Transwell membranes were then stained with crystal violet. For quantification, the average number of migrating cells per field was assessed by counting 5 random fields under a phase-contrast microscope (Leica, German) at 200x magnification.

### 2.7. Tube Formation Assay

The HUVECs tube formation assay was performed with an ECMatrix assay kit, following the manufacture's protocol. Briefly, HUVECs cells were seeded in 6-well culture plates at a density of 2 × 10^5^ cells/mL. After overnight incubation, HUVECs were treated with indicated concentrations of PZH for 24 hours in normoxic conditions, harvested and diluted to 2 × 10^5^ cells/well in 200 *μ*L media, seeded into 1 : 1 ECMatix gel (v/v) coated 48-well plates, and incubated at 37°C under normoxic/hypoxic conditions for 6 hours. Tubes were imaged using a phase-contrast inverted microscope (Leica, German) at 100x magnification. The degree of tube formation was quantified by counting the number of tube structures in four randomly chosen fields without overlap.

### 2.8. ELISA

The secretion level of VEGF was detected by ELISA kit according to the manufacturer's protocol. The results were expressed as fold change in the concentration of VEGF relative to that of cells in normoxia.

### 2.9. RT-PCR Assay

Total RNA was extracted from cultured cells using the Trizol reagent (TakaRa). cDNA was synthesized from 2 *μ*g of total RNA with a Reverse Transcription Kit (TakaRa). The primers used in the RT-PCR reactions were the following: (forward primer) 5′-CTC AAA GTC GGA CAG CCT CA-3′ and (reverse primer) 5′-CCC TGC AGT AGG TTT CTG CT-3′ for HIF-1*α*, (forward primer) 5′-TTT CTG CTG TCT TGG GTG CAT TGG-3′ and (reverse primer) 5′-TCT GCA TGG TGA TGT TGG ACT CCT-3′ for VEGF, and 5′-CTC CAG GGC TTC TTG GTT TTC C-3′ and (reverse primer) 5′-TTT CAC CAT CTG GTT GGC TGG C-3′ for *α*-tubulin. The PCR products were separated and visualized on an agarose gel stained with ethidium bromide under UV transillumination.

### 2.10. Western Blot

Cultured cells were lysed in a buffer containing 40 mM Tris-Cl, 10 mM EDTA, 120 mM NaCl, 0.1% NP-40, and a protease inhibitor cocktail (CALBIOCHEM). Protein concentration was determined using a bicinchoninic acid (BCA) assay. Denatured proteins were separated by SDS/polyacrylamide gel electrophoresis (PAGE) and transferred to a PVDF membrane (MILLIPORE). The membrane was incubated with 5% skim milk in TBS containing 0.1% Tween-20 for 1 hour at room temperature and probed with the appropriate antibodies. The blots were developed using an ECL Prime Detection Kit (Amersham Pharmacia Biotech).

## 3. Results

### 3.1. Intrinsic Cytotoxicity of PZH in HUVECs

**Figure 1:**
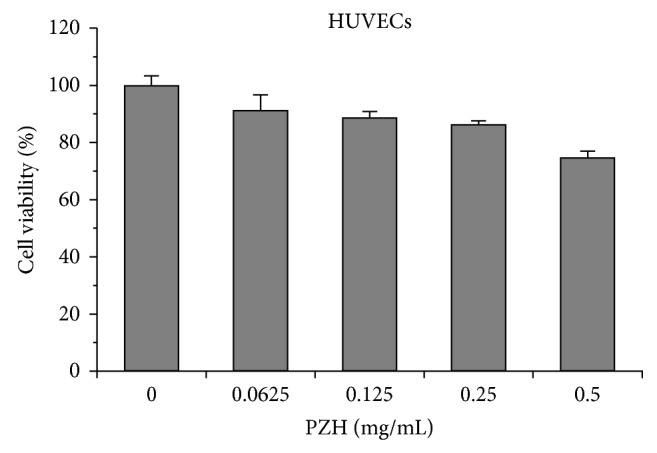
The intrinsic cytotoxicity of PZH in HUVECs. HUVECs cells were treated with the indicated concentrations of PZH for 24 h. Cell viability was determined by MTT assay. The data were normalized to the viability of control cells. Data are averages with SD (error bars) from at least three independent experiments.

The lowest dose (0.0625 mg/mL) of PZH was not cytotoxic to HUVECs, and higher doses (0.125 and 0.25 mg/mL) only decreased cell viability by 11.36% and 13.69%, respectively (Figure 1). To investigate angiogenesis inhibition effects of PZH, treatment concentrations of 0.0625–0.25 mg/mL were chosen.

### 3.2. PZH Inhibited Hypoxia-Induced Migration of HUVECs

**Figure 2:**
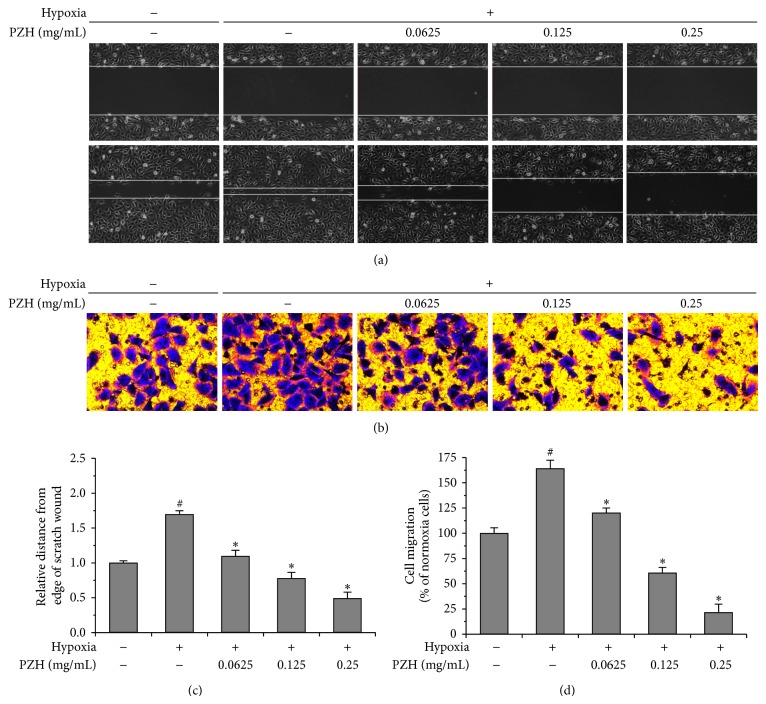
Effect of PZH on the migration in HUVECs. After treatment with the indicated concentrations of PZH for 24 h, the migration pattern of HUVECs was observed using phase contrast microscopy, wound-healing, and transwell migration assays. (a) A confluent monolayer of HUVEC was scratched in the middle with a pipette tip and the scratched area covered by cells that had migrated after exposure to normoxia or hypoxia for 12 h was measured; photographs are at 100x magnification. Images are representative of three independent experiments. (b) Transwell migration assays of HUVECs. Images are at 100x magnification. Images are representative of three independent experiments. (c) Migration distance after wound relative to the control at time of wound. ^#^A significant difference compared with the normoxic control (*P* < 0.05). ^*^A significant difference compared with the hypoxic control (*P* < 0.05). (d) Measurement of the average number of migrating cells per field was assessed by counting 5 random fields. Statistics are expressed as the mean ± SD. ^#^A significant difference compared with the normoxic control (*P* < 0.05). ^*^A significant difference compared with the hypoxic control (*P* < 0.05).

Since endothelial cell migration is a pivotal step for angiogenesis, we performed wound-healing and transwell migration assays in HUVECs pretreated with indicated concentrations of PZH (Figure 2) [32]. Dose dependent attenuation of hypoxia-induced increase in migration rates into wounds was observed (Figures 2(a) and 2(c)). Hypoxia alone increased transwell migration by 1.64-fold as compared to normoxia while intervention with PZH lowered hypoxia-induced increased transwell migration rates to below baseline in a dose dependent manner (Figures 2(b) and 2(d)).

### 3.3. PZH Inhibited Hypoxia-Induced Tube Formation of HUVECs

**Figure 3:**
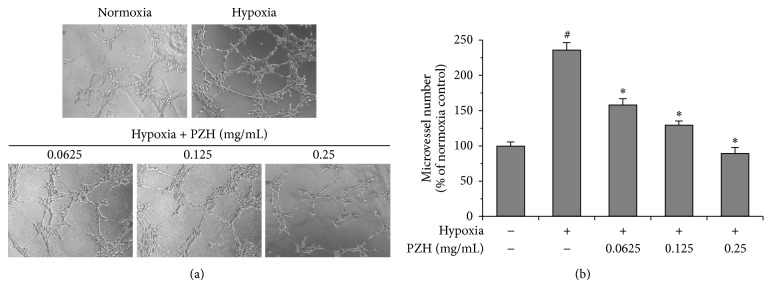
Effects of PZH on tube formation in HUVECs. (a) HUVECs cells were treated with PZH for 24 h, and then collected HUVECs were cultured in Matrigel coated plates. Representative photomicrographs, at 200x magnification, of three independent experiments are shown. (b) The representative histogram of the area covered by the tube network was quantitated using Image-Pro Plus software. ^#^A significant difference compared with normoxic control (*P* < 0.05). ^*^A significant difference compared with hypoxic control (*P* < 0.05).

PZH blunted increased capillary-like networks due to hypoxic conditions in a dose dependent manner as well (Figure 3).

### 3.4. PZH Inhibited Hypoxia-Induced Expression of HIF-1*α* and VEGF-A in HCT-8 Cells and HUVECs

**Figure 4:**
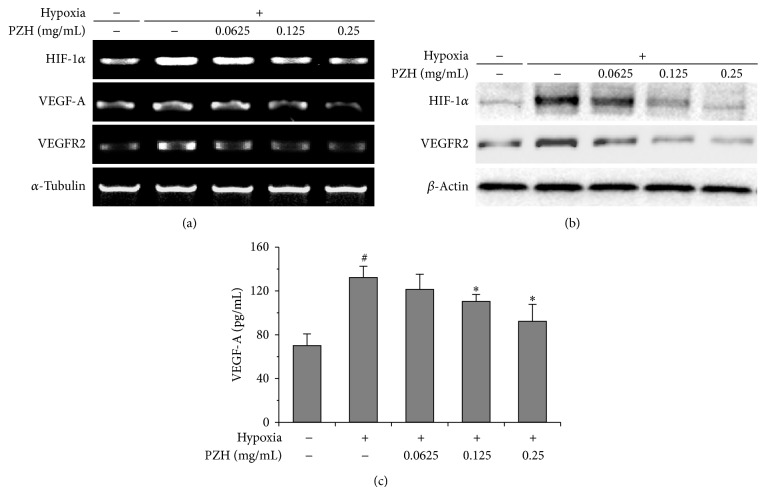
Effects of PZH on HIF-1*α*, VEGF-A, and VEGFR2 expression in HUVECs. (a) mRNA expression levels of HIF-1*α* were analyzed by RT-PCR, and *α*-tubulin was used as a loading control. (b) Protein expression levels of HIF-1*α* and VEGFR2 were analyzed by Western blot, and *β*-actin was used as a loading control. (c) Secreted VEGF protein was analyzed by ELISA. ^#^A significant difference compared with normoxic control (*P* < 0.05). ^*^A significant difference compared with hypoxic control (*P* < 0.05).

**Figure 5:**
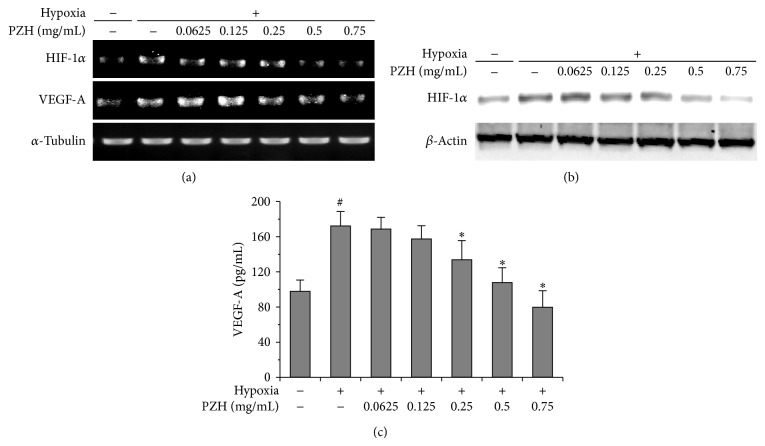
Effects of PZH on the expression of HIF-1*α* and VEGF-A in HCT-8. (a) mRNA expression levels of HIF-1*α* were analyzed by RT-PCR, and *α*-tubulin was used as a loading control. (b) Protein expression levels of HIF-1*α* were analyzed by Western blot, and *β*-actin was used as a loading control. (c) Secreted VEGF protein was analyzed by ELISA. ^#^A significant difference compared with normoxic control (*P* < 0.05). ^*^A significant difference compared with hypoxic control (*P* < 0.05).

HIF-1*α* is known to be stabilized during hypoxia driving VEGF expression promoting angiogenesis [33]. We therefore examined whether PZH could influence the expression patterns of HIF-1*α* and VEGF-A in HCT-8 and HUVECs cells exposed to hypoxia. PZH suppressed HIF-1*α* protein levels as well as mRNA expression in both HUVECs and HCT-8 cells (Figures 4(a), 4(b), 5(a), and 5(b)). Moreover, PZH inhibited hypoxia-induced secretion of VEGF-A in both cell lines as well (Figures 4(c) and 5(c)). These results suggest that downregulated HIF-1*α*/VEGF-A pathway by PZH could inhibit the hypoxia-stimulated migration and tubular formation of endothelial cells.

## 4. Discussion 

HIF-1*α*, a transcription factor, has been demonstrated to be associated with most hypoxic solid tumors and is consistently upregulated in various types of cancers and promotes tumorigenesis through angiogenesis [19, 34, 35]. Aberrant expression of VEGF is known to be a key regulator in hypoxia-induced angiogenesis. Although PZH is known to inhibit VEGF expression via multiple cellular pathways, the effect of PZH on hypoxia-induced VEGF expression and the regulation of HIF-1*α* activity by PZH are not well understood [31]. In this study, we have demonstrated that VEGF, a potent hypoxia-induced angiogenic factor, and HIF-1*α*, the transcription factor essential for VEGF transcriptional activation, can be regulated by PZH in HCT-8 and HUVECs. We further revealed that PZH directly suppresses hypoxia-stimulated endothelial migration and tube formation, which is also accompanied by decreased HIF-1*α* and VEGF expression levels in PZH-treated HUVECs. A recent report showed that loss of hypoxia-responsive HIF-1*α* in endothelial cells results in impaired angiogenesis. This impairment is a direct result of the disruption of an autocrine loop acting through HIF-1*α* regulation of hypoxia-induced VEGF expression [33]. Based on our observations in this project, PZH may play a direct role in the degradation of HIF-1*α* and subsequent VEGF signaling in colorectal cancer cells and endothelial cells, leading to the inhibition of hypoxia-induced tumor angiogenesis, which is consistent with our previous study that PZH displays antitumor angiogenesis activity in the colorectal cancer mouse xenograft model [31].

Interestingly, many drugs may enhance vascularization in normal injured tissue while doing the reverse in tumor. Although there are no reports directly demonstrating the effects of Pien Tze Huang (PZH) on vascularization in the normal injured tissue, PZH is believed to be able to promote would healing because it has long been used to clinically treat traumatic injuries. In addition, recently it has been reported that* Panax notoginseng* saponins (PNS), one of major components in PZH, exerted a significant therapeutic effect on a complex disease condition featuring concomitant presentation of lung carcinoma and myocardial ischemia in mouse, where PNS was able to inhibit tumor angiogenesis and meanwhile promote myocardial ischemia-induced angiogenesis [36]. Therefore, we speculate that PZH probably also bidirectionally regulates angiogenesis: enhancing vascularization in normal injured tissue while doing the reverse in tumors. Although Yang et al. further proposed that PNS bidirectionally regulated angiogenesis through modulating the expression of proangiogenic miR-18a in a tissue specific manner, they did not address why PNS displayed opposite regulation of angiogenesis and related miRNA expression in different tissues. The possible mechanisms mediating the bidirectional angiogenic activity of PNS and other drugs could be the difference of microenvironment between tumors and normal tissues.

It has been shown that tumor blood vessels differ morphologically from their normal counterparts, which display inappropriate growth and abnormal proliferation and apoptosis in endothelial cells, insufficient pericyte recruitment, enhanced leakiness, decreased blood flow, and abnormalities in the basement membrane [37, 38]. Moreover, gene expression analysis of endothelial cells derived from human blood vessels of normal and malignant colorectal tissues revealed that forty-six genes were specifically elevated in tumor-associated endothelium [39]. Furthermore, hypoxia is a common characteristic feature of all rapidly growing solid tumors, which may trigger the activation of tumor-related signaling pathways such as HIF-1*α* [40]. However, the precise mechanisms of how drugs exert bidirectional angiogenic function remain largely unclear. These intriguing and challenging questions must be addressed by future studies before these drugs can be developed as better antiangiogenic agents for cancer treatment.
